# Immediate processing of erotic stimuli in paedophilia and controls: a case control study

**DOI:** 10.1186/1471-244X-13-88

**Published:** 2013-03-19

**Authors:** Benedikt Habermeyer, Fabrizio Esposito, Nadja Händel, Patrick Lemoine, Markus Klarhöfer, Ralph Mager, Volker Dittmann, Erich Seifritz, Marc Graf

**Affiliations:** 1 Department of General and Social Psychiatry, Psychiatric University Hospital, Zürich, Switzerland; 2 Department of Forensic Psychiatry, University of Basel, Basel, Switzerland; 3 Department of Medicine and Surgery, University of Salerno, Baronissi, (SA), Italy; 4 Department of Medical Radiology, MR-Physics, University of Basel, Basel, Switzerland; 5 Clinic for Affective Disorders and General Psychiatry, Psychiatric University Hospital, Zurich, Switzerland

**Keywords:** Paedophilia, Event related fMRI, Erotic stimulation

## Abstract

**Background:**

Most neuroimaging studies investigating sexual arousal in paedophilia used erotic pictures together with a blocked fMRI design and long stimulus presentation time. While this approach allows the detection of sexual arousal, it does not enable the assessment of the immediate processing of erotically salient stimuli. Our study aimed to identify neuronal networks related to the immediate processing of erotic stimuli in heterosexual male paedophiles and healthy age-matched controls.

**Methods:**

We presented erotic pictures of prepubescent children and adults in an event related fMRI-design to eight paedophilic subjects and age-matched controls.

**Results:**

Erotic pictures of females elicited more activation in the right temporal lobe, the right parietal lobe and both occipital lobes and erotic pictures of children activated the right dorsomedial prefrontal cortex in both groups. An interaction of sex, age and group was present in the right anteriolateral oribitofrontal cortex.

**Conclusions:**

Our event related study design confirmed that erotic pictures activate some of the brain regions already known to be involved in the processing of erotic pictures when these are presented in blocks. In addition, it revealed that erotic pictures of prepubescent children activate brain regions critical for choosing response strategies in both groups, and that erotically salient stimuli selectively activate a brain region in paedophilic subjects that had previously been attributed to reward and punishment, and that had been shown to be implicated in the suppression of erotic response and deception.

## Background

Paedophilia is a matter of great public interest and often evokes emotional discussions in mass media. It is difficult to estimate the prevalence of paedophilia as both paedophilic offenders and victims often prefer not to identify themselves. From surveys of the general population we know that approximately 12% of men and 17% of women report experiencing sexual abuse in their childhood [1]. These figures underline the potential impact of this disease on society. According to DSM IV-TR, paedophilia is defined by two main criteria: firstly persistent sexual fantasies, urges or behaviour involving sexual activity with prepubescent children and secondly that these people have acted on these urges, or that these urges or fantasies caused marked distress or interpersonal difficulties [2].

Findings from neuropsychological studies on paedophilia are heterogeneous. Whilst a lower IQ [3], educational difficulties [4] and a higher rate of left-handedness [5] indicate rather generalised brain dysfunction, other studies suggest more specific alterations like focal weaknesses in frontal-executive [6,7] and/or temporal-verbal [8] skills or even a more deliberate response style and greater self-monitoring in paedophilic subjects [9,10]. Furthermore revealed research on personality traits in paedophilia various findings like impaired interpersonal functions, impaired self-awareness, disinhibitory traits, sociopathy and a propensity for cognitive distortions [11]. In summary, it can be stated that the neurobiology of paedophilia remains incomplete.

In recent years, researchers have increasingly addressed the neuronal underpinnings of sexual arousal in order to better understand sexual behaviour. Stoléru et al. [12,13] and Redouté et al. [14,15] were the first to propose a neurophenomological model that disentangled the cognitive, emotional, motivational and physiological components of sexual arousal. Two recently published quantitative meta-analyses on sexual cue reactivity [16,17] underline that distinctive subcomponents of sexual arousal can be reliably localised by neuroimaging techniques.

Based on the fMRI results about sexual arousal in healthy controls it was natural to transfer that approach to paedophilic subjects in order to better understand the cognitive and behavioural processes in paedophilia. In heterosexual paedophilia fMRI studies have demonstrated the altered processing of erotic visual stimuli in the dorsomedial prefrontal cortex (DMPFC), amygdala and hippocampus [18]. The latter study also reports that erotic pictures of adults induced a stronger activation of the hypothalmus, the periaqueductal grey and the dorsolateral prefrontal cortex (DLPFC) in healthy controls than in heterosexual paedophilic subjects. While this study highlights differences between controls and paedophilic subjects other studies report similar activation patterns for the respective erotic conditions. For example Schiffer et al. [19] show that erotic pictures of girls induced the same activation of limbic structures such as the amygdala, the cingulate gyrus or the hippocampus in heterosexual paedophilia as erotic pictures of women in a control group. However this study also found an additional activation of the DLPFC in the heterosexual paedophilic group alone. Research has also been carried out on homosexual paedophilia. Again in both paedophilic and control subjects, a common network relating to sexual activation was found, comprising the occipitotemporal and prefrontal cortex [20]. However, only paedophilic subjects showed activated subcortical regions such as the thalamus, the globus pallidus and the striatum during erotic stimulation. Another study on homosexual paedophilia describes differential activation of the right orbitofrontal cortex [21].

Most of the above-mentioned studies indicate that prefrontal brain regions might be related to paedophilia, but the results and structures involved differ from study to study. Considering these rather heterogeneous results, the findings from a recent study by Ponseti et al. [22] appear surprising. These authors propose an fMRI based classification procedure for heterosexual and homosexual non-paedophilic and paedophilic subjects with 95% accuracy. Unlike previous studies, these authors describe activation in regions known to be involved in the processing of erotic stimuli such as the caudate nucleus, cingulated cortex, insula, fusiform gyrus, temporal cortex, occipital cortex, thalamus, amygdala and cerebellum but not in the prefrontal cortex.

Except for the study of Walter et al. [18] all of the above-mentioned studies that describe a prefrontal involvement in paedophilia used relatively long presentation times ranging from 19.2 - 38.5 s and blocked fMRI designs.

One critical shortcoming of long presentation times is that different cognitive processes, such as sustained attention or self-referential processes might also take place and somehow interfere with the target process. Earlier fMRI studies on sexual arousal in normal subjects showed that with shorter presentation times of sexually arousing pictures specific neuronal networks and brain processes can be addressed. Static presentation for 8.75 s as used by Moulier et al. [23] demonstrated for example that the initiation and low levels of penile tumescence are controlled by frontal, parietal, insular and cingular cortical areas. In accordance with these results, Ferretti et al. [24] showed that longer presentation times (> 30 s) induce sexual arousal and penile erection whilst shorter presentation times (< 3 s) induce arousal without erection. A stimulation time of 5 s even allowed to distinguish specific sexual emotional effects from more general emotional effects [25]. A study comparing both types of fMRI-designs in the visual processing of erotic stimuli in healthy volunteers [26] provides strong support for the use of fast stimulation time, suggesting an event related design. The authors proposed that event related designs might be an alternative to blocked designs if the core interest is the detection of networks associated with the immediate processing of erotic stimuli.

Besides being more useful for the detection of these networks, short presentation times also offer other potential benefits as long presentation times in paedophilia are even more problematic than in healthy controls. Paedophilic subjects are mostly recruited after coming into conflict with the legal authorities as a result of their sexual preferences and this may increase a tendency to suppress erotic arousal or dissimulate sexual attraction to the pictures of children presented. The latter point has been used to explain some of the differences between ratings of erotic salience and brain response observed in some of the aforementioned studies [19,21]. As presentation time becomes shorter, deliberate manipulation may become more difficult and the immediate processes following the perception of target stimuli could become visible.

In our study we aim to identify the neuronal networks related to immediate processing of erotic stimuli in heterosexual paedophilic subjects recruited from a forensic outpatient setting. Based on previous neuropsychological research and recent fMRI studies we predicted a differential activation in the prefrontal cortex in paedophilic subjects.

## Methods

### Subjects

Behavioural and functional magnetic resonance imaging data were acquired from 16 male right-handed subjects. All participants in this study were adults. Written informed consent was obtained from all subjects before participation in the study.

The subjects in the paedophilic (N = 8) group were recruited from an outpatient cognitive behavioural group therapy at the department of forensic psychiatry in Basel, Switzerland. The heterosexual paedophilic subjects fulfilled DSM-IV-TR criteria for exclusive type (attracted only to prepubescent children) not limited to incest heterosexual paedophilia. Three of the subjects had previously molested prepubescent children, the other five had been convicted because of the possession of large quantities of explicit internet child pornography. Each participant’s sexual orientation and preference for prepubescent erotic stimuli was assessed in a clinical interview, the Multiphasic Sex Inventory (MSI) [27] and additionally verified by the clinical record and the court file. For all subjects, neither the interview nor the record indicated other comorbid paraphilias. None of the subjects had admitted paedophilia prior to the contact with the legal authorities.

Heterosexual control subjects (N = 8) were recruited from an advert on the University Hospital notice board. A clinical evaluation of all subjects revealed no other psychiatric, neurological or medical conditions. The local Ethics Committee of the University of Basel, Switzerland approved the study.

### Stimulation and paradigm

The study design was adopted from Bühler et al. [26]. Visual stimuli were generated and displayed on a personal computer using the Neurobehavioral Systems (NBS) software package Presentation® 12.2. Stimuli were presented via fMRI-compatible digital video goggles (NordicNeuroLab, Bergen, Norway).

**Figure 1:**
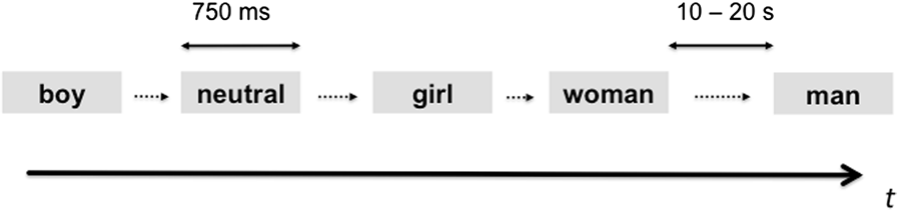
**FMRI paradigm.** We presented erotic pictures of adults (women/men) and children (girls/boys) interspersed with neutral control pictures. Pictures from each category were presented for 750 ms with a jittered interstimulus interval ranging from 10–20 s.

While inside the scanner, subjects were presented with various types of pictures in an event related design. We presented 10 pictures from each category (erotic pictures of boys, girls, men, women or neutral control pictures) for 750 ms with a jittered interstimulus interval that varied randomly between 10 – 20 s in steps of full seconds (Figure 1).

In each picture, only one person of one of the above-mentioned categories was displayed in bathing clothes in front of a plain-coloured background. All pictures showed faces and the complete corpus. Secondary sexual characteristics were clearly visible in the pictures showing adults but were clearly absent in the pictures depicting prepubescent children. We avoided photographs of adolescents and applied a biological rather than a legal cut off to make the pictures of adults easily distinguishable from those of children. Before inclusion into the paradigm rated the study subjects the pictures and only the pictures with the highest ratings on a visual analogue scale were included. Neutral pictures showed simple objects like e.g. a small boot in front of the same background.

In order to control attention during the passive fMRI task we used a previously applied procedure to assure attentive observation of the presented pictures [28]: Before the scanning procedure, subjects were instructed to attentively observe the pictures and told that we would check their attention by asking them to identify the pictures after the scanning session. Immediately after the fMRI session we presented the slides that they had seen interspersed with similar pictures, which had not been presented during the experiment. Subjects then had to decide which pictures they had seen during the fMRI stimulation.

### FMRI acquisition and analysis

Images were acquired using a 3 T MRI scanner (Verio, Siemens Healthcare, Erlangen, Germany) equipped with a standard radio frequency head coil. First a T1-weighted high-resolution data set that covered the whole brain was acquired using a three-dimensional MPRAGE (magnetization-prepared rapid acquisition gradient echo) sequence with a repetition time (TR) of 2.00, an isotropic spatial resolution of 1.0 mm^3^, and an echo time (TE) of 3.4 ms. T2* weighted functional images were recorded using echoplanar imaging with a TR of 2500 ms, an isotropic spatial resolution of 3×3×3 mm^3^ and a TE of 30 ms (FoV 228; matrix 76; spacing between slices: 0.51 mm; interslice time: 69 ms). Altogether 152 volumes with 36 image slices with a thickness of 3 mm were obtained.

### Image pre-processing and statistical analysis

Image time-series were processed using the BrainVoyager QX 2.3.0 software package (Brain Innovation, Maastricht, The Netherlands). Pre-processing included head motion correction, slice scan time correction, temporal high pass filtering and removal of linear trends. Using the results of the image registration with anatomical scans, the functional image-time series were then warped into Talairach space and resampled into 3 mm isotropic voxel time-series. Normalized images were smoothed using a 6.00 mm isotropic Gaussian kernel.

For first-level analysis, a General Linear Model (GLM) analysis was applied with separate subject z-normalized predictors fitted to z-normalized voxel time-courses from all data sets. The orthogonal predictors of interest in the design matrix were: “boys”, “girls”, “men”, “women” and “neutral”. For second-level analysis, the GLM fits (beta weights) were assessed in a 3-way-ANOVA with two within subject factors (sex: female vs. male; age: child vs. adult) and one between subject factor group (paedophilia vs. control). We assessed the main effects of, and the interaction between, these factors at the voxel level and obtained maps that thresholded at the significance level of 5% (corrected for multiple comparison). To obtain this correction, an uncorrected statistical threshold was initially applied to each of the F-maps at p = 0.005, and a cluster-level threshold correction procedure based on Monte Carlo simulations [29] was applied in order to determine the minimum cluster size below which any activation was to be discarded. This procedure yielded a minimum cluster size of 10 voxels (corresponding to a minimum cluster extent of 280 mm) for the F-maps of the interaction of all factors, 12 voxel (297 mm) for the factor age and 19 voxels (482 mm) for the factor sex.

The anatomical labelling of active clusters was defined on the basis of the peak voxel coordinates, using the Talairach Daemon (http://www.talairach.org) [30,31].

Active clusters resulting from the voxel-level ANOVA were further defined as Regions of Interests (ROI). Data from those ROIs were extracted and analysed in order to understand the direction of the effects of the ANOVA. To that end, we calculated linear contrasts for the different factors, i.e. sex (female > male), age (child > adult) and the interaction of the factors (girl > woman) separately for both groups. In order to further specify the interaction of all factors (sex, age, group) we extracted BOLD %-signal-change from the respective ROI and averaged the BOLD%-signal-change separately for the groups.

## Results

### Demographics

Sexual orientation in the control group was heterosexual with a preference for mature women. In the paedophilia group all subjects were heterosexual and attracted exclusively by prepubescent stimuli. The mean age of the paedophilic subjects was 48.25 (standard deviation: 9.15) years and mean IQ 116 (12.12). In the control groups, the mean age was 46.25 (8.35) years and IQ 124.88 (13.91). Student t-tests showed no significant difference between these groups (Table 1). In the paedophilic group 75.4% of the pictures were correctly identified immediately after the scanning session. In the control group 77.5% of the pictures were correctly identified. Again a group difference was excluded by *t*-test.

**Table 1:** **Mean age, IQ and rate of the correctly indentified pictures (behavioural control) with standard deviation (SD) and *T***-**test for both groups**

	**Paedophilia**		**Controls**		***T*-test**
	**mean**	**SD**	**mean**	**SD**	**p**
Age (years)	48.25	9.15	46.25	8.38	0.35
IQ (points)	116.00	12.12	124.88	13.91	0.21
Recognized pictures (%)	74.25	22.25	77.38	14.93	0.39

None of the results reaches statistically significant differences.

### FMRI

#### Voxel level ANOVA

**Figure 2:**
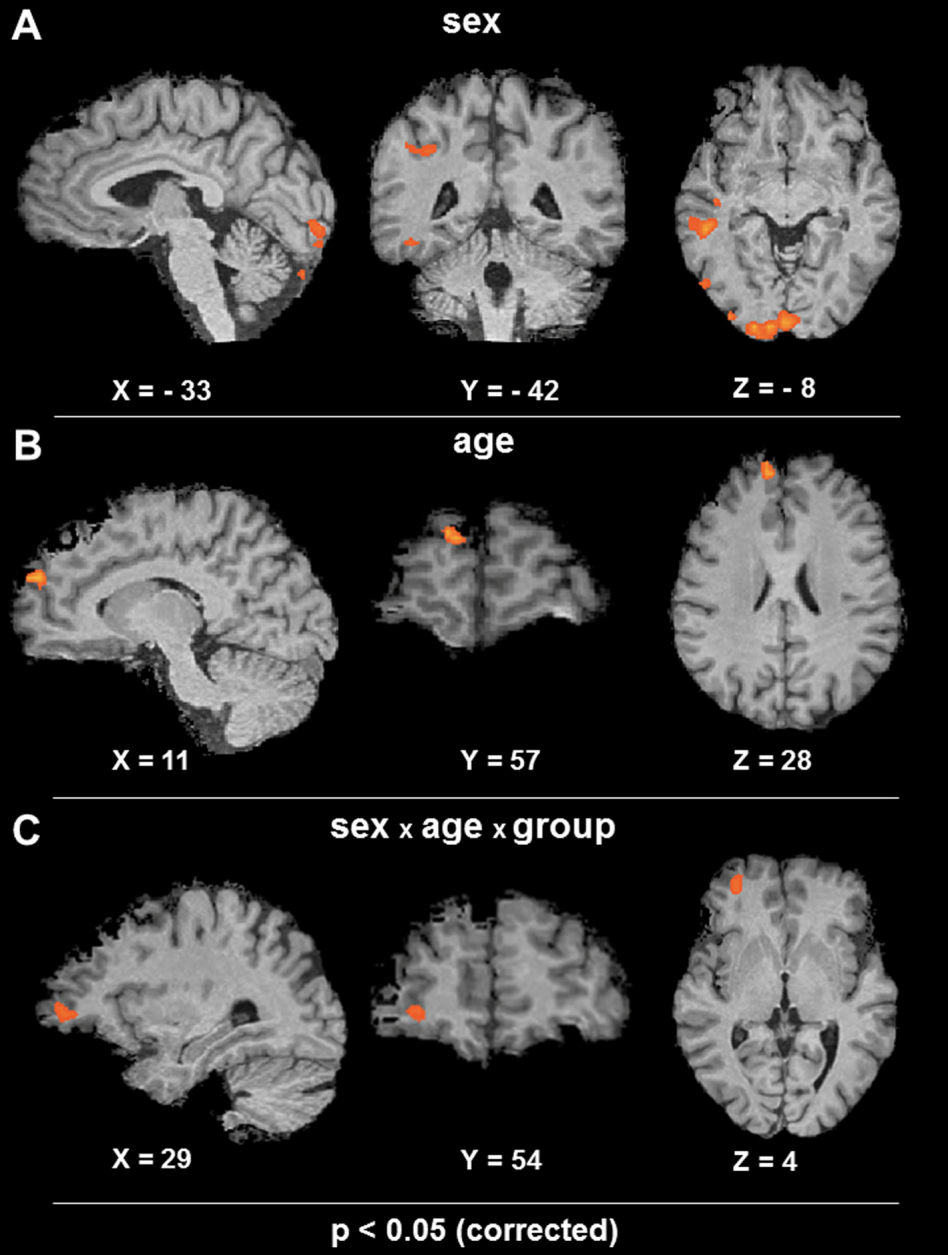
**Whole brain ANOVA.** Active voxels showed a main effect of the factor sex (female/male) in the right middle temporal gyrus, right fusiform gyrus, right inferior and middle occipital gyrus and right parietal lobe. In the left hemisphere the inferior occipital gyrus displayed active voxels (**A**). Furthermore we found bilateral activation in the cerebellum. The factor age (adult/child) showed an activation of the right dorsomedial prefrontal cortex (**B**). In the right lateral orbitofrontal cortex a significant interaction of sex, age and group was detected (**C**). Significant voxels in A-C represent effects at a corrected p-level of p < 0.05. X, Y, Z corresponds to the Tailarach coordinates.

We found a significant interaction of the factors sex, age and group (p < 0.05, corrected) in the middle frontal gyrus (Figure 2, Table 2). We furthermore found significant main effects for the factors sex and age outside of the middle frontal gyrus, i. e. in brain areas that didn`t show a significant interaction of all factors.

**Table 2:** Regions that showed a significant effect in the voxel wise ANOVA

**voxel level ANOVA**	**X**	**Y**	**Z**	**Side**	**Lobe**	**Structure**	**BA**	**Cluster size (mm**^**3**^**)**
sex	51	−34	−5	R	Temporal Lobe	Middle Temporal Gyrus	21	1289
	45	−17	−12	R	Temporal Lobe	Middle Temporal Gyrus	21	504
	41	−42	34	R	Parietal Lobe	Supramarginal Gyrus	40	820
	41	−78	−11	R	Occipital Lobe	Fusiform Gyrus	19	1510
	32	−85	8	R	Occipital Lobe	Middle Occipital Gyrus	19	421
	9	−94	−8	R	Occipital Lobe	Inferior Occipital Gyrus	17	3526
	6	83	−29	R	Cerebellum			773
	−33	−90	−15	L	Occipital Lobe	Inferior Occipital Gyrus	18	989
	−20	−86	−20	L	Cerebellum			340
	−25	−85	−32	L	Cerebellum			369
age	11	57	28	R	Frontal Lobe	Superior Frontal Gyrus	9	433
sex × age × group	29	54	4	R	Frontal Lobe	Middle Frontal Gyrus	10	428

X, Y, Z corresponds to the Tailarach coordinates (BA = Brodmann area; R = right, L = left).

A significant main effect of the factor sex (female, male) was present at a corrected threshold of p < 0.05 in the right middle temporal gyrus, right fusiform gyrus, right inferior and middle occipital gyrus and right parietal lobe. In the left hemisphere, the inferior occipital gyrus was also active. We also found bilateral activation in the cerebellum.

The factor age revealed an activation of the right dorsomedial prefrontal cortex (p < 0.05, corrected).

For the factor group, no significant activation was found at the latter threshold, but again this finding is restricted to brain areas that did not show a significant interaction of all factors.

### ROI analysis

**Figure 3:**
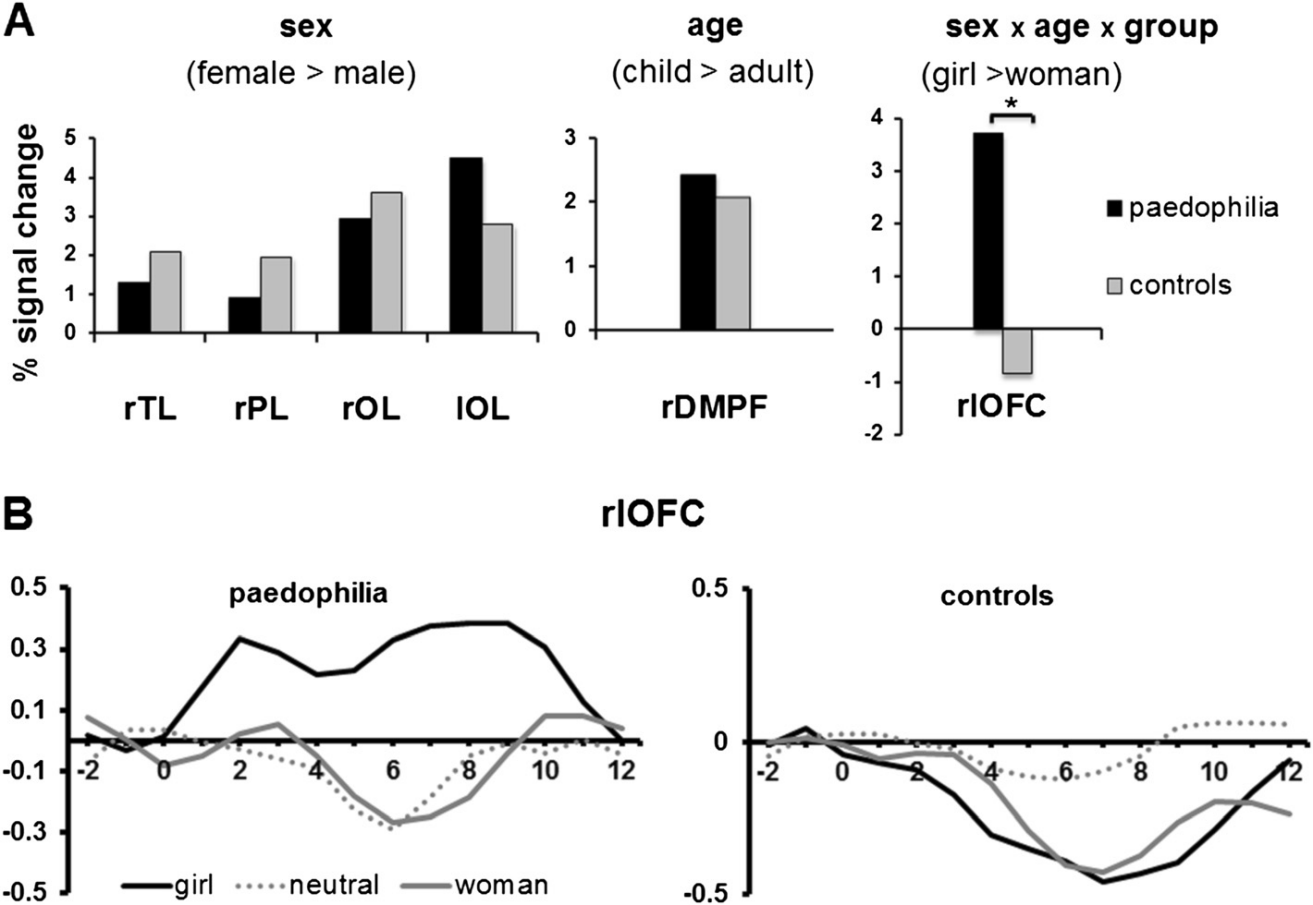
**ROIs analysis (A). The ROI female > male contrast level for the ROIs extracted from the ANOVA of the main effect of sex displayed a greater activation for female pictures in all active ROIs (rTL = right temporal lobe, rPL = right parietal lobe, rOL = right occipital lobe, lOL = left occipital lobe).** The child > adult contrast confirmed that the main effect of age is based on a stronger activation due to pictures of children in both groups (rDMPFC = right dorsomedial prefrontal cortex). ROI analysis of the cluster showing an interaction of all factors revealed a significant difference (* = p < 0.05) for the contrast girl > woman, with activation in paedphilic subjects and deactivation in control subjects (rlOFC = right lateral orbitofrontal cortex). Event related averaging (**B**). BOLD %-signal-change in the rlOFC only illustrated a strong activation due to erotic pictures of girls in paedophilia.

The linear contrasts of these ROIs confirmed that, in both groups, the main effect of the factor sex was based on a higher activation due to female pictures and the main effect of the factor age on a stronger activation for pictures of children. ROI analysis clarified that in the ROI that became significant for the interaction of all factors, only paedophilic subjects showed activation in the girl condition while controls showed a deactivation in this condition (Figure 3A).

Event related averaging of BOLD %-signal-change for all conditions and all groups in that ROI illustrated a strong activation due to erotic pictures of girls in paedophilic subjects that was absent for all other picture conditions in both groups (Figure 3B).

## Discussion

Visual presentation of erotic pictures has been used to address the underpinnings of erotic stimulation in paedophilia [19-22,28,32]. Results proved heterogeneous, which might partially be explained by the detection of non-specific secondary processes in blocked designs with long stimulus presentation time.

Our study focuses on the immediate response to erotic pictures. Like previous studies using a short presentation time and an event-related fMRI design [22,26,33] we also found that female erotic pictures elicited more activation in the right temporal lobe, the right parietal lobe, both occipital lobes and the cerebellum in both heterosexual groups. This finding indicates that these regions respond to erotic visual stimulation in an immediate fashion, e.g. activation can be detected even after a short presentation time. This finding highlights that this activation seems independent from non-specific processes such as sustained attention. Furthermore there was no difference between BOLD response to female erotic pictures in both heterosexual groups.

Pictures of children elicited a differential activation regardless of sex in the right DMPFC. This is appears to be a very interesting finding as a crucial role in the critical evaluation of stimuli and for the preparation of response strategies has been attributed to the DMPFC [34]. All study subjects were well aware of the fact that our study was about paedophilia. We would expect that erotic pictures of children would be recognised as a stimulus of special interest in that context. Both groups are also likely to be aware that this condition is specifically attached to socially unaccepted sexual preference and behaviour. These assumptions might explain why we found a specific activation of the DMPFC in both groups, independent from the sex of the presented pictures. In the DMPFC these pictures are apparently not differentiated by preferred sex or specific erotic salience but are rather perceived as a category requiring specific attention. This assumption would nicely fit with the findings from Walter et al. [35] indicating that activation of the DMPFC is not related to specific processes such as the processing of erotic content, but rather to more general processes like attention. In line with our findings, Ponseti et al. reported [33] that the medial orbitofrontal cortex showed a stronger response to erotic pictures than neutral pictures, but this response was also independent from individual sexual preference, as it was present in heterosexual male subjects with both pictures of male and female erotic stimuli. In contrast to earlier studies showing a group effect and a different activation pattern in the prefrontal cortex after presentation of erotic pictures of children, our findings indicate that in the context of immediate processing of visual erotic stimuli a considerable overlap in the processing of these stimuli is present. Considering that both groups rely on the same neuronal stimulus evaluation and response preparation networks, this finding seems rather less surprising.

In contrast to the earlier studies that presented pictures for more than 30 s, we focus on the immediate response to visual stimuli. Many of the previous studies were conducted with forensic inpatients while we only included outpatients and most of our paedophilic study group were convicted due to their consumption of illegal internet pornography. Other studies [36,37] already demonstrated that social functioning and neurocognitive capabilities are apparently better in these subjects than in forensic inpatients with hands-on delinquency. In this context it has to be stressed that the IQ of the paedophilic participants can be considered extremely high especially in view of the already mentioned findings from Cantor et al. [3]. Hence we cannot exclude that selection of the study subjects contributed to our findings and that the generalisability of our results might be limited.

Brain response in the right lateral oribitofrontal cortex (OFC) was shown to interact with the factors sex, preferred age and group. Mean group betas and event related averaging confirmed a specific activation of the right lateral OFC due to erotic pictures of prepubescent girls in heterosexual paedophilic subjects only whereas all other conditions in the paedophilic group and all conditions in the control group displayed a deactivation of the lateral OFC. Further support for a deviant activation in the right lateral OFC can be found in an fMRI study by Walter et al. [18] that revealed a reduced activation for nude stimuli of female adults in paedophilic subjects.

FMRI research has already confirmed that OFC can be activated by visual inputs [38]. According to Kringelbach et al. [39] the OFC is a nexus for sensory integration. Phillips et al. [40] stated that OFC is related to the identification of the emotional significance of a stimulus and to the production of affective states in response to that stimulus. These authors furthermore suggested that, together with the amygdala, insula, ventral striatum and ventral anterior cingulated, the OFC is also involved in the automatic regulation of emotional responses. Hence, different processes such as the modulation of autonomic reactions, learning, prediction, decision making and emotional and reward-related behaviours are processed in the OFC. According to an fMRI study by Sescousse et al. [41] the anterior part of the lateral OFC responds more strongly to more abstract rewards such as monetary gain, while the posterior lateral OFC responds more strongly to more basic rewards, for example erotic stimulation. These findings were in line with a study by Kringelbach and Rolls [42] describing a posterior anterior distribution of primary (e.g. sexual stimulation) and more abstract secondary (monetary gain) reinforcers. They suggested that the anterior part of the lateral OFC is considered to be a more recent structure, phylogentically speaking, and might therefore be related to the processing of more abstract reinforcers like money.

Findings from our study underline the notion that the OFC plays a critical role in evaluating reinforcers that might provoke behavioural changes [42,43]. This is even more interesting, as it has been shown that the anterior lateral OFC responds more to punishment while the anterior medial part of the OFC responds to reward [44,45]. In line with this idea, the specific group effect in the right lateral OFC might indicate that the specific emotional significance of the stimuli was only detected in paedophilic subjects. Erotic pictures of children provoked a brain response resembling that of punishment in paedophilic subjects. According to the literature, the right anterior lateral OFC is not only associated with punishment but also with cues indicating a behavioural shift. This significance for behavioural adoption has been highlighted in recent reviews on the OFC [39,43,46]. This is interesting as healthy controls have demonstrated that the lateral OFC became active when subjects tried to suppress erotic responses to visual erotic stimulation [47] or in deception [48]. Our results would therefore indicate that immediately after the presentation of erotic pictures of children, a brain region known to be involved in evaluating emotional salience became active in paedophilic subjects. This activation might be related not only to the regulation of emotional states or autonomic responses but also to behavioural changes like suppression or deception of emotional responses.

The findings from our study point to a network perspective of the immediate processing of visual erotic stimulation. This notion would be in line with the so-called orbital and medial prefrontal cortex [49]. In this network two likely interacting systems became active or inactive while processing the visual stimuli. Even after a short presentation of visual stimuli the DMPFC showed an increased activation in response to the target condition of our study (erotic pictures of children). It is most likely that this finding is related to attentional processes, which are evoked in both study groups. We further found a specific activation due to the respective erotic condition in the anterior lateral OFC in paedophilic subjects. Activation in that region might indicate evaluation of emotional salience and the reward value of that stimulus. Furthermore this activation might indicate the intent to modify behaviour and to dissimulate or deceive erotic responses.

Assessing the immediate processing of erotic pictures using fMRI might be a useful tool for probing emotional engagement towards the presented stimuli in future studies. These results could also be therapeutically relevant as most cognitive behavioural treatment approaches aim to reduce cognitive distortions and the denial of the implications of paedophilic behaviours and to increase the awareness of problematic attraction to children [50] rather than to change the sexual preference. The often-reported tendency of paedophilic subjects to dissimulate and minimise the dramatic impact on potential victims of child abuse, might arise from similar processes, which aim at suppressing emotional distress. Interestingly Ponseti et al. [22] did not find such an activation in their study sample, which in contrast to our sample deliberately stated paedophilic sexual deviance.

## Conclusion

Our fMRI study confirms the findings of previous studies concerning the processing of visual erotic stimuli. Furthermore we were able to demonstrate that the dorsomedial prefrontal cortex is specifically engaged in processing erotic pictures of children regardless of the study group. This activation appears to represent the evaluation of relevance, which is apparently not linked to sexual orientation per se but rather to the study context. In addition we have made some new findings regarding the immediate processing of visual erotic stimulation in paedophilia, as we found an immediate activation of the brain regions involved in evaluating emotional salience and reward, and engaged in the regulation of emotional responses. This activation might be related to the suppression or deception of erotic responses.

## Abbreviations

OFC: Orbitofrontal cortex; DMPFC: Dorsomedial prefrontal cortex; DLPFC: Dorsolateral prefrontal cortex; MPRAGE: Magnetization prepared rapid acquisistion gradient echo; GLM: General linear model, ROI: region of interest.

## Competing interests

The authors declare that they have no competing interests.

## Authors’ contributions

BH, RM, MG, VD and ES conceived and designed the study. BH, NH, RM, PL and MK realized the study design and acquired the data. BH, FE, NH performed data analyses. BH and FE interpreted data and wrote the manuscript. BH, FE, NH, PL, MK, RM, VD, ES and MG contributed in the drafting and revision of the manuscript for important intellectual content. All authors read and approved the final manuscript.

## Pre-publication history

The pre-publication history for this paper can be accessed here:

http://www.biomedcentral.com/1471-244X/13/88/prepub
